# The resilience of emergency and critical care nurses: a qualitative systematic review and meta-synthesis

**DOI:** 10.3389/fpsyg.2023.1226703

**Published:** 2023-10-02

**Authors:** Shuyang Liu, Yu Zhang, Yue Liu, Peng Han, Yugang Zhuang, Jinxia Jiang

**Affiliations:** ^1^Emergency Department, Shanghai Tenth People's Hospital, School of Medicine, Tongji University, Shanghai, China; ^2^Community health service center of Pengpu new village street of Shanghai Jing’an District, Shanghai, China

**Keywords:** resilience, emergency and critical care, nurses, systematic review, qualitative

## Abstract

**Background:**

Due to the unique work environment, emergency and critical care departments nurses face high job pressure, often resulting in burnout and a high turnover rate. Public health emergencies such as the Corona Virus Disease 2019 pandemic tend to exacerbate these problems further. Therefore, improving the resilience of nurses is crucial to enhance their retention rates.

**Objective:**

This systematic review and meta-synthesis of qualitative studies on the resilience of emergency and critical nurses were conducted to provide a reference for clinical managers to develop strategies for improving the resilience of nurses.

**Methods:**

Following databases were searched for relevant studies: CINAHL Plus, Elsevier, Cochrane Library, Embase, Medline, OVID, Pubmed, Science Direct, LWW and Web of Science, China National Knowledge Network (CNKI), Wanfang Database (CECDB), VIP Database, and Sinomed. Google Scholar and Opengrey were used to search for gray literature. The literature search period was from the establishment of the database to April 2023. The systematic review of qualitative studies followed the Joanna Briggs Institute (JBI) approach, including critical appraisal using the JBI Checklist and synthesis through meta-synthesis. Confidence of evidence was assessed with JBI’s ConQual process.

**Results:**

A total of 12 articles were identified, with 59 main results and 9 new integrated categories. Also, 3 themes, i.e., risk factors, protective factors, and personal growth, and 9 sub-themes, i.e., working pressure, negative emotion, an organizational issue, active learning, sense of occupational benefit, social support, self-cognition and regulation, learn to adapt, and self-actualization, were formed.

**Conclusion:**

The resilience of emergency and critical care nurses depends on various factors. Managers should prioritize the mental health of nurses and implement measures to enhance their resilience through social support, team building, and psychological capital development. Additionally, management models can be updated based on domestic and international experience to improve nurses’ job involvement, optimize nursing quality, and promote the advancement of the nursing profession.

## Introduction

The emergency and critical care (ECC) departments have always been at the forefront of medical practice, serving as the exclusive hospitalization option for patients with diverse and critical conditions requiring immediate attention. Consequently, nurses working in these departments encounter heightened levels of stress compared to other departments due to the unique professional environment they operate within and the inherent demands of their occupation (Wu, 2015). The significance of the ECC departments has been widely acknowledged by the general public. During public health emergencies, such as the Corona Virus Disease 2019 (COVID-19) pandemic, ECC nurses bear primary responsibility. Despite infection risks, they made remarkable contributions by screening suspected and confirmed cases, implementing infection control measures, enhance measures to contain the epidemic, monitoring vital signs, collecting specimens, providing non-invasive and invasive ventilation support, administering mechanical circulation assistance (ECMO), and caring for critically ill patients (Han et al., 2022). In addition, ECC nurses, who spend the most time beside the patient’s bed and have the highest level of contact with the patient, play an indispensable role as the backbone of the department. An analysis of their clinical role in intensive care revealed that their collaboration with physicians is critical to reducing emergency department waiting times, enhancing patient satisfaction, reducing mortality, and saving health care costs. The benefits of this approach surpass those of solo physician work (Woo et al., 2017).

However, unpredictable factors and high levels of uncertainty govern the working atmosphere in the ECC departments, making it an inferior working environment compared to other departments. The workload is heavy, the pace is fast and the intensity is elevated, increasing the risk of burnout for ECC nurses. Burnout is a psychological syndrome that occurs due to long-term work-related stressors, resulting in an inability to cope with emotional stress or depletion of energy and resources, leading to feelings of failure, and exhaustion (Schooley et al., 2016). A study conducted in Spain reported that one-third of emergency and critical care nurses experienced high burnout levels (Cañadas-de la Fuente et al., 2018). There is a strong link between job burnout and the turnover intention, as evidenced by multiple studies (Hämmig, 2018; Lee et al., 2021; Özkan, 2022). With an aging population, increasing demand for healthcare services, and the threat of infectious diseases, the global nurse shortage is becoming ever more severe (Peters, 2023). According to existing studies, older age groups utilize emergency and critical care services more frequently and require additional care due to complex chronic diseases accompanied by atypical symptoms. The need for labor in such fields is also expected to increase (Angus et al., 2000; Armoon et al., 2021; Wilhelms and Wilhelms, 2021). The issue of nurse burnout is particularly acute in this context. The shortage of ECC nurses leads to longer patient wait times, overcrowding in emergency departments, lower patient satisfaction, more nurse–patient conflicts, and difficulties with the ambulance allocation (Gorman, 2019). In fact, it has been suggested that reducing the number of ECC nurses could compromise patient safety and clinical outcomes (Adams et al., 2019). Furthermore, such loss would lead to an increased financial burden on hospitals and intensified pressure on the remaining staff (Adams et al., 2019). During the previous COVID-19 pandemic, emergency and intensive care nurses faced a significantly higher risk of mental health issues and job burnout due to understaffing, surging workload, limited medical resources, a highly contagious disease with high mortality rates, and social stigma associated with infection (Jiang et al., 2022). Due to these challenges, the job satisfaction of nurses has plummeted, and turnover rates soared (Lopez et al., 2022). However, literature (Han et al., 2022) suggests a close link between the mental health of nurses, nursing care quality, and patient clinical outcomes. Nurses with higher levels of resilience are more likely to stay in their jobs longer and are less likely to experience burnout. Training nurses to gain the necessary professional skills in the ECC departments requires significant time and effort, making nurse retention a top priority.

Resilience is a multi-dimensional concept that lacks a unified definition. It generally refers to an individual’s ability to adapt well to adversity, pressure, and trauma (Han et al., 2023). The current definition of resilience involves two aspects, i.e., the stressor and the individual’s ability to adapt (White et al., 2008). With the emergence of positive psychology, studies pertaining to resilience have increasingly been applied to diverse cohorts. Among these, the exploration of resilience in nurses has expanded both the scope and application domain of resilience research, while also offering valuable insights for further in-depth investigations within professional contexts (Lin and He, 2018). A review suggested that resilience in the field of nursing is a complex dynamic process that enables nurses to actively adapt to workplace stressors, avoid psychological harm, and consistently provide safe, high-quality patient care (Han et al., 2023). A study have shown that higher nurse resilience is associated with lower job burnout, indicating a significant negative correlation between the two (Guo et al., 2018). Resilient nurses can better handle adversity and trauma during public health emergencies through self - motivation (Jiang et al., 2022). Enhanced resilience in nurses is crucial for increasing retention, enhancing career satisfaction, improving nursing quality, and promoting career development. Because qualitative research can more profoundly explore the emotional experience of respondents and the deepest aspects of their spirit than other methods, and because of regional and cultural limitations, a single qualitative study may not be adequately representative of all ECC nurses. Therefore, the purpose of this study is to systematically review and synthesize qualitative studies on resilience in ECC nurses from different cultures. This will provide nursing managers with a valuable reference for developing more effective programs that can bridge the gap between clinical practice and research.

## Materials and methods

### Objective

The objective of this review is to identify factors affecting resilience in ECC nurses, and reveal the authentic requirements of ECC nurses in cultivating resilience. This review focuses on the following questions: How do ECC nurses understand their own resilience and the factors that influence it? What strategies can managers employ to enhance the resilience of ECC nurses?

### Design

The meta-synthesis method was adopted to conduct a systematic review of qualitative research. Based on a comprehensive understanding of the philosophical foundation and methodology of meta-synthesis, we conducted repeated readings of the included studies to extract themes and implied meanings. Subsequently, we synthesized themes with similar connotations, established new categories, and integrated novel findings. By synthesizing new results, deeper and more substantive explanations of specific phenomena can be made, thus providing more influential and persuasive final conclusions (Sandelowski et al., 2007). This review used the Preferred Reporting Items for Systematic Review and Meta-Analysis (PRISMA) (Moher et al., 2009) as the basis for reporting the review. The results were reported using the Enhancing Transparency in Reporting the Synthesis of Qualitative Research (ENTREQ) (Tong et al., 2012).

### Search strategy

The following 10 databases were searched: CINAHL Plus, Elsevier, Cochrane Library, Embase, Medline, OVID, Pubmed, LWW, Science Direct, Web of Science, and in addition, four Chinese databases were searched: CNKI, VIP, Sinomed and Wanfang. Google Scholar and Opengrey were used to search for grey literature. The search method was almost identical for each database, that is, keywords are used to define the target literature and Boolean operators are used to connect them. Search terms included: nurs*, emergency*, acute, acute care, critical, critical care, ICU, intensive care unit, intensive care, resilient*, bounce*, elasticity, resistance, tenacity, toughness, mental toughness, and recovery. The search period spanned the time the database was established until April 2, 2023. Also, references to relevant literature were tracked to avoid omissions.

### Eligibility criteria and study selection

**Figure 1:**
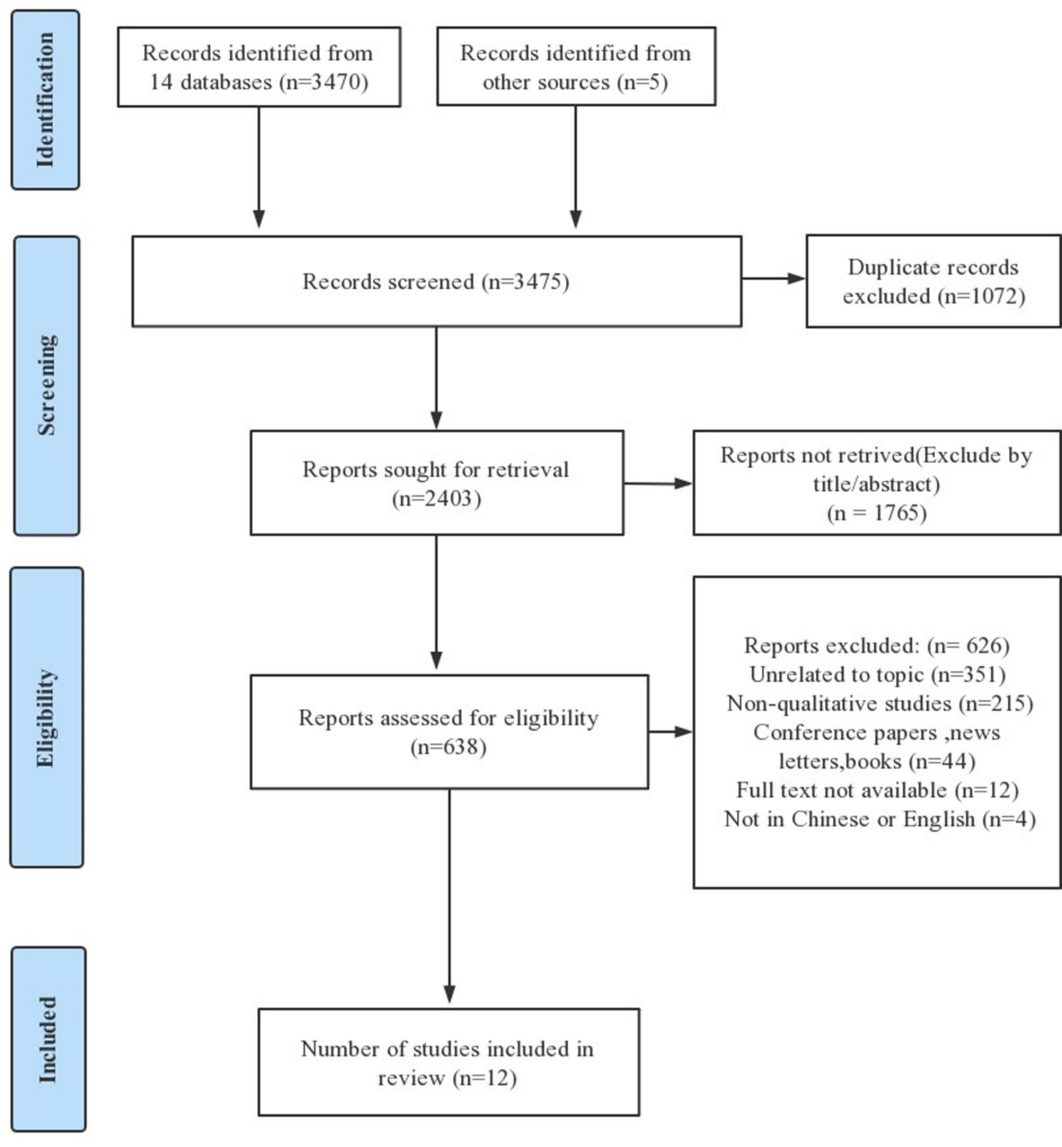
Article screening process.

The inclusion and exclusion criteria are shown in Table 1. After a systematic search, 3,475 literature sources were found. The document management software Endnote was used to organize them. After removing 1,072 duplicates and scanning the titles of the remaining sources, 1765 unrelated sources were removed. Finally, after screening for inclusion criteria through abstracts and full texts, only 12 sources remained for quality evaluation. Two researchers independently performed the literature screening process and cross-checked the results. A third researcher was consulted in case of disagreement, and finally, a consensus was reached on the results. Figure 1 shows the search results, selection, and the inclusion process according to the Preferred Reporting Items for Systematic Reviews and Meta-Analyses (PRISMA) 2020 statement (Page et al., 2021).

**Table 1:** Inclusion and exclusion criteria of the article.

Inclusion criteria	Exclusion criteria
P (Population): Nurses working in emergency and critical care departments	Full text article is not available
I (Interest of phenomena): The comprehension and experience of resilience among ECC nurses in the face of adversity and the factors influencing their resilience.	Non-chinese or non-English article
Co (Context): There are certain positive experiences for ECC nurses after adversity or trauma.	Article with repeated publication or incomplete data
S (Study design): Qualitative research refers to a systematic and subjective approach to describe life experience and assign meaning to it, includes articles that employ qualitative research methods such as phenomenology, ethnography, grounded theory, and action studies.	Article with a grade of C after quality evaluation

### Quality assessment

Two researchers trained in the systematic evidence-based nursing course independently evaluated the quality of the included articles by using the Australian JBI Centre for Evidence-based Health Care Qualitative Research Quality Evaluation criteria (Lockwood et al., 2015), which consists of 10 items, including whether the research methodology is consistent with its philosophical basis, data collection method, data analysis method, and result interpretation, as well as the typicality of the research object, the researcher’s own factors and ethical review. Each item is assessed as follows: 2 for “yes,” 1 for “unclear,” and 0 for “no”/“not applicable.” Finally, the scores for each question were summarized and converted to a percentage. Literature with a score of >70% was included. Two researchers agreed upon the final score, and no literature was eliminated after quality evaluation. Table 2 shows the results of the quality evaluation.

**Table 2:** Evaluation of methodological quality.

Included studies	①	②	③	④	⑤	⑥	⑦	⑧	⑨	➉	Results (%)
Jiang et al. (2022)	Y	Y	Y	Y	Y	N	N	Y	Y	Y	16/20 (80%)
Ang et al. (2019)	Y	Y	Y	Y	Y	N	N	Y	Y	Y	16/20 (80%)
Mealer et al. (2012)	Y	Y	Y	U	Y	N	N	Y	Y	Y	15/20 (75%)
Hodges et al. (2008)	Y	Y	Y	Y	Y	N	N	Y	U	Y	15/20 (75%)
Shi et al. (2020)	Y	Y	Y	Y	Y	N	N	Y	U	Y	15/20 (75%)
Li et al. (2021)	Y	Y	Y	Y	Y	N	U	Y	U	Y	16/20 (80%)
Hancock et al. (2020)	Y	Y	Y	Y	Y	N	U	N	Y	Y	15/20 (75%)
Yuan et al. (2022)	Y	Y	Y	Y	Y	N	U	Y	Y	Y	17/20 (85%)
Marey-Sarwan et al. (2022)	Y	Y	Y	U	Y	U	U	Y	Y	Y	17/20 (85%)
Conolly et al. (2022)	Y	Y	Y	Y	Y	N	U	Y	Y	Y	17/20 (85%)
Huang et al. (2021)	Y	Y	Y	Y	Y	U	Y	Y	Y	Y	19/20 (95%)
Jiang et al. (2020)	Y	Y	Y	N	Y	U	U	Y	U	Y	15/20 (75%)

JBI Australian Centre for Evidence-based Health Care Qualitative research Quality evaluation criteria (Lockwood et al., 2015): ① Is there congruity between the stated philosophical perspective and the research methodology? ② Is there congruity between the research methodology and the research question or objectives? ③ Is there congruity between the research methodology and the methods used to collect data? ④ Is there congruity between the research methodology and the representation and analysis of data? ⑤ Is there congruity between the research methodology and the interpretation of results? ⑥ Is there a statement locating the researcher culturally or theoretically? ⑦ Is the influence of the researcher on the research, and *vice-versa*, addressed? ⑧ Are participants, and their voices, adequately represented? ⑨ Is the research ethical according to current criteria or, for recent studies, and is there evidence of ethical approval by an appropriate body?➉ Do the conclusions drawn in the research report flow from the analysis, or interpretation, of the data?

### Data extraction and meta-synthesis

After carefully analyzing the literature, the two researchers extracted important information such as author, country, year, study purpose, data collection and analysis methods, subject characteristics and numbers, and study results. A third researcher was consulted in case of any disputes. Each finding in the original study was assigned a level of credibility (Munn et al., 2014): an outcome was deemed unequivocal (U) when it directly related to what was described in the article; an outcome was considered credible (C) when it appeared plausible or could be inferred from the content of the article; if there was no correlation between the content of the article and the result, or if the result was not reported in the article, it was considered as unsupported (NS). Two researchers rated each result for confidence, cross-checked it, and then asked a third researcher to resolve disagreements, if any.

In line with interpretive philosophy, meta-synthesis integrates multiple qualitative research results to generate new concepts and meanings based on postmodernism’s multi-faceted understanding and interpretation of a phenomenon (Walsh and Downe, 2005). Furthermore, it aims at providing comprehensive and reliable evidence, embodying the concept of evidence-based nursing and promoting rational resource utilization (Walsh and Downe, 2005). Drawing on an understanding of qualitative research methodology and philosophical concepts, each finding presented in the literature undergoes iterative refinement and analysis. Results with similar meanings are then categorized into new groups, which are subsequently integrated to form novel findings. Finally, all researchers engaged in discussions and confirmed the synthesized results.

### ConQual-assessment of confidence of evidence

The ConQual system, which evaluates and grades meta-synthesized bodies of evidence from qualitative studies, was constructed by the JBI Center for Evidence-Based Health Care in 2014 (Munn et al., 2014). The system assesses the credibility and dependability of integrated evidence, resulting in a ConQual-based quality rating of high, medium, low or very low. When assessing dependability and credibility, meta-synthesized evidence is assumed to be high quality and evaluated based on three aspects of credibility and five of dependability. Dependability focuses on the quality of the original studies included in the analysis, while credibility considers whether the integrated results are consistent with the supporting data. The ConQual system scores for this review are shown in Table 3.

**Table 3:** ConQual system scores and the specific reason.

Synthesized findings	Type of research	Dependability	Credibility	ConQual score	Comments
Risk factors	Qualitative research –phenomenological, descriptive	Downgrade one level - Moderate∗	Remains unchanged**	Moderate	The findings came from 8 papers *Downgraded one level as the majority of studies (7 out of 8) scored 3 on questions related to the appropriateness of the conduct of the study **Remains unchanged as all findings unequivocal
Protective factors	Qualitative research–phenomenological, descriptive	Downgrade one level - Moderate∗	Remains unchanged**	Moderate	The findings came from 10 papers *Downgraded one level as the majority of studies (8 out of 10) scored 3 on questions related to the appropriateness of the conduct of the study **Remains unchanged as all findings unequivocal
Personal growth	Qualitative research–phenomenological, grounded theory	Downgrade one level - Moderate∗	Remains unchanged**	Moderate	The findings came from 8 papers *Downgraded one level as the majority of studies (6 out of 8) scored 3 on questions related to the appropriateness of the conduct of the study **Remains unchanged as all findings unequivocal

## Results

The 12 qualitative studies included in the study scored between 70 and 95%, indicating a medium to high level of quality, according to the Australian Centre for Evidence-Based Health Care quality assessment tool. None of the studies, however, provided clear information about the cultural background and values of the researchers. In addition, one study did not acknowledge the subjects’ own words as the basis for their conclusions (Hancock et al., 2020), and only one study mentioned the researcher’s influence (Huang et al., 2021). The four articles lacked clarity on whether they received approval from an ethics committee (Ang et al., 2019; Jiang et al., 2020; Shi et al., 2020; Li et al., 2021). The representativeness and typicality of the research objects in the three articles were not outstanding (Mealer et al., 2012; Jiang et al., 2020; Marey-Sarwan et al., 2022), which could affect the dependability of the meta-integrated evidence body. However, since each level of evidence in qualitative studies is unequivocal, credibility remains unchanged. Eventually, the ConQual level was found to be moderate.

**Figure 2:**
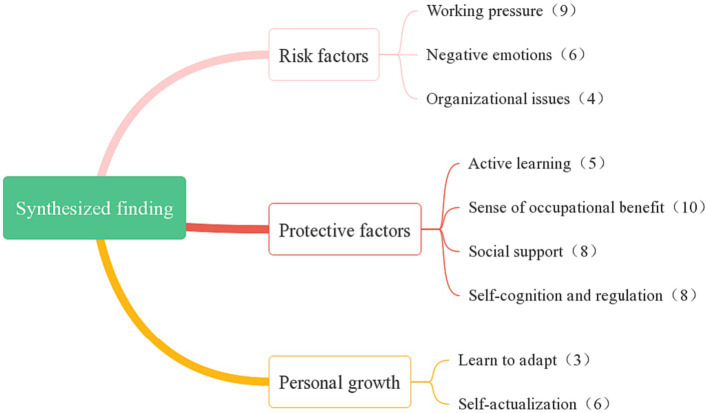
Meta-synthesis results 421 and categories.

This review included 12 qualitative studies involving a total of 205 subjects. Seven used the phenomenological approach (Hodges et al., 2008; Jiang et al., 2020, 2022; Shi et al., 2020; Li et al., 2021; Marey-Sarwan et al., 2022; Yuan et al., 2022) 1 used grounded theory (Ang et al., 2019), 1 described qualitative research (Marey-Sarwan et al., 2022), and 3 did not specify methodology (Mealer et al., 2012; Hancock et al., 2020; Conolly et al., 2022). Most studies (10 of the 12) used semi-structured interviews to collect data, while the remaining 2 used focus group interviews (Ang et al., 2019; Hancock et al., 2020), and diaries (Shi et al., 2020), respectively. Furthermore, 8 studied the resilience of ECC nurses in public health emergencies such as that induced by COVID-19 (Jiang et al. 2020, 2022; Shi et al., 2020; Huang et al., 2021; Li et al., 2021; Conolly et al., 2022; Marey-Sarwan et al., 2022; Yuan et al., 2022). In addition, 6 studies were from China (Jiang et al., 2020; Shi et al., 2020; Huang et al., 2021; Li et al., 2021; Jiang et al., 2022; Yuan et al., 2022), 2 from the United States (Hodges et al., 2008; Mealer et al., 2012), and 1 from Canada (Hancock et al., 2020), the United Kingdom (Conolly et al., 2022), Israel (Marey-Sarwan et al., 2022), and Singapore (Ang et al., 2019), respectively. The time span was from 2008 to 2022. After analysis, 59 clear findings were obtained and combined into 3 comprehensive results with the addition of 9 new categories. The specific extraction results of the included studies are shown in Table 4. The results of the meta-synthesis, categories, and the number of unequivocal findings are shown in Figure 2.

**Table 4:** Characteristics of qualitative studies.

Authors and year	Origin	Aim	Methodology	Results
Jiang et al. (2022)	China	To explore the resilience of nurses in the emergency department of Shanghai during the COVID-19 pandemic	Using a phenomenological approach to qualitative research, semi-structured in-depth interview via wechat video call. The sample size was 17, including 6 males and 11 females. Age range: 23–46	Three themes emerged: (1) Risk factors (2) Protective factors (3) Altruistic drive
Ang et al. (2019)	Singapore	To explore the different ways of resilience of acute and intensive care nurses	Using grounded theory of qualitative research, face-to-face interview. The sample size was 18, including 3 males and 15 females. Age range: 24–68	Three themes emerged: (1) Self-efficacy (2) Coping style (3) Work attitude
Mealer et al. (2012)	United States	To explore the psychological resilience and post-traumatic stress disorder of ICU nurses in the United States	Qualitative research and semi-structured telephone interview were used The sample size was 27, including 1 male and 26 females. Age range: 35–59	Four themes emerged: (1) World outlook (2) Social relations (3) Cognitive flexibility (4) Self-balance
Hodges et al. (2008)	United States	Explore the professional resilience of Baccalaureate ICU nurses	Using an interpretive phenomenological approach to qualitative research, face-to-face semi-structured in-depth interview. The sample size was 11, including 1 male and 10 females, with an age range of 23–31.	Three themes emerged: (1) Learning the milieu (2) Conscious integration (3) Growth
Shi et al. (2020)	China	To explore changes in the resilience of ICU nurses in Wuhan during the COVID-19 outbreak	Using a phenomenological approach to qualitative research. Diary as source material. The sample size was 9, including 2 males and 7 females. Age range: 26–35	Three themes emerged: (1) Stress period (2) Buffer period (3) Recombination period
Li et al. (2021)	China	To explore the experience of front-line nurses in designated hospitals dealing with COVID-19 from the perspective of resilience	Using a phenomenological approach to qualitative research, semi-structured in-depth interview via wechat video call. The sample size was 12 from ICU, including 4 males and 8 females. Age range: 23–43. ICU nursing age ranges from 0.5 to 10 years	Three themes emerged: (1) Multiple risk factors (2) Multiple protective factors (3) Multidimensional adaptation strategies
Hancock et al. (2020)	Canada	Explore burnout and ethical dilemmas in the ICU team to build resilience	Using qualitative research and Focus group interview. The sample size was 21. Male accounted for 80%, those who have worked for 0–5 years, 6–15 years and more than 16 years account for 37.1, 31.4 and 31.4%, respectively	Three themes emerged: (1) Organizational problems (2) High-pressure environment (3) Lack of team experiences
Yuan et al. (2022)	China	To explore the track of resilience of first-line nurses in Wuhan during the novel coronavirus epidemic	Phenomenological methods of qualitative research were used through semi-structured phone interviews. The sample size was 12. Age range: 29–36. Including 2 males and 10 females.	Three themes emerged: (1) Challenges and difficulties (2) Overcome difficulties (3) Personal growth
Marey-Sarwan et al. (2022)	Israel	To explore resilience and coping strategies among nurses during the COVID-19 pandemic	Using phenomenological methods of qualitative research, face-to-face semi-structured interview and Zoom software are adopted. The sample size was 18. Age range: 31–53.	Three themes emerged: (1) Adjust occupational demands and family life (2) Influencing factors of resilience and nurses’ coping strategies (3) The application of metaphorical language
Conolly et al. (2022)	United Kingdom	Explore the building of nurse resilience during the COVID-19 pandemic	Using narrative in-depth interviews of qualitative research, Sample size:27. Mainly female and only three were from minority groups.	Three themes emerged: (1) Resilience is a badge of honor (2) The spur of resilience (3) Take pride in resilience
Huang et al. (2021)	China	Explore the resilience of frontline nurses in China during the COVID-19 pandemic	Descriptive qualitative research was used and in-depth interviews were conducted via cell phone. The sample size was 23. The average age is 30 years, and the average length of service is 9 years.	Three themes emerged: (1) Initial negative emotions (2) Positive mental state after 1–2 weeks (3) Influencing factors
Jiang et al. (2020)	China	Explore the resilience of nurses during the COVID-19 pandemic	Using the phenomenological method of qualitative research and face-to-face and semi-structured interviews, the sample size was 10, all female and age range: 24–40.	Four themes emerged: (1) Resilience cognition (2) Resilience regulation (3) Adapt to the situation (4) Self-actualization

### Synthesized finding 1: risk factors

The synthesized finding includes three specific categories reflecting risk factors: working pressure, negative emotions, and organizational issues. These specific categories were extracted from eight articles (Hancock et al., 2020; Shi et al., 2020; Huang et al., 2021; Li et al., 2021; Conolly et al., 2022; Jiang et al., 2022; Marey-Sarwan et al., 2022; Yuan et al., 2022).

### Working pressure

Working pressure is a crucial risk factor affecting the resilience of ECC nurses. While appropriate pressure can motivate people, excessive pressure can lead to adverse outcomes such as job burnout, compassion fatigue, and resignation (Poncet et al., 2007). In their study, Hancock et al. (2020) argued that workplace violence and complex nurse–patient relationship were unique sources of stress for ECC nurses compared with other medical staff. In addition, factors such as heavy workload and intense work pace increased the psychological burden of nurses. During the previous COVID-19 pandemic, ECC departments were greatly affected, which caused increased difficulty and stress to medical staff due to material shortages, understaffing, and physical fatigue. “*We spend an average of six hours constantly working and on duty,”* said an ICU nurse involved in the COVID-19 response (Li et al., 2021). Another nurse said: “*The isolation ward is understaffed, and there are so many patients. I hand out medicine and check each patient more than 20 times and always worry that I have forgotten something. I feel exhausted by the end of the day (Yuan et al.,**2022**)*.” A nurse said she had to “juggle” between the role of the social worker, physical therapist, and nutrition manager because there was no one to answer due to the shortage of nursing staff (Marey-Sarwan et al., 2022). In addition, the physiological fatigue caused by wearing protective clothing and its influence on the operation also increased the psychological pressure of nurses: “*I have to take blood from all patients between 3 a.m. and 6 a.m. Because of the fogging of goggles and protective gloves, it is difficult for me to operate, and I cannot finish all the tasks on time (Huang et al.,**2021**).”*

### Negative emotions

In addition to working pressure, negative emotions hinder nurses’ resilience. During the previous COVID-19 pandemic, ECC nurses had to cope with intrusive thoughts like anxiety, fear, and loneliness caused by the fear of the unknown, excessive workload, concern for family members, and patients’ negativity. In their study, Shi et al. (2020) reported that among the study subjects, four nurses were married and expressed concern about their children, who were their top priority, and they felt guilty for not fulfilling their family role. Some even reported avoiding seeing elderly family members (Marey-Sarwan et al., 2022). Many nurses spoke of their unprecedented fear due to the rapid spread of the disease: “*Many of my colleagues around me are getting infected, and I am afraid that I am next. It seems dangerous to touch anyone who is sick (Jiang et al.,**2022**).*” Because of the impact of the pandemic, people had to keep social distancing themselves from each other. One nurse said: “*I feel more lonely than ever, I work most of the time and cannot go out to see friends and family (Marey-Sarwan et al.,* *2022**).”* In their study, Huang et al. (Huang et al., 2021) reported that some nurses mentioned that they also felt dispirited because of the patients’ depressed mood.

### Organizational issues

Public health emergencies like COVID-19 highlighted issues within the organization, such as supply and staff shortages and a lack of emergency preparedness, a nurse said: “*We even avoid drinking water or going to the bathroom to save on protective clothing.*” Some isolation wards had to redeploy nurses from other departments due to the staff shortage, some of whom have previous experience in intensive isolation wards (Conolly et al., 2022). However, inadequate preparation, lack of communication with the new team, and unfamiliarity with the department culture in the new environment can lead to feelings of exclusion and helplessness, “*I felt really unsafe in this weird environment and there was nobody I could turn to for support. I’m not too familiar with a lot of the procedures, so I’m scared that I might end up delaying patients*,” said one transfer nurse (Conolly et al., 2022). At the same time, nurses in the original department felt impatient with newcomers (Jiang et al., 2022): “*New nurses are not familiar with various procedures. Now, I have to teach them and do my own thing. I am exhausted.*” In addition, ICU nurses identified issues with management education and training, inadequate contingency plans for health emergencies like COVID-19, and policies out of touch with the public as areas requiring attention (Hancock et al., 2020).

### Synthesized finding 2: protective factors

The synthesized finding includes four specific categories reflecting protective factors: active learning, sense of occupational benefit, self-cognition and regulation, social support. These specific categories were extracted from 10 articles (Hodges et al., 2008; Mealer et al., 2012; Jiang et al., 2020, 2022; Shi et al., 2020; Huang et al., 2021; Li et al., 2021; Conolly et al., 2022; Marey-Sarwan et al., 2022; Yuan et al., 2022).

### Active learning

Active learning can help nurses quickly adapt to adversity and foster resilience. Learning has a crucial role in the adaptability of new nurses seeking knowledge and the initiative of experienced nurses exploring the unknown during crises like COVID-19 (Hodges et al., 2008; Li et al., 2021). One new nurse said: “*I used to keep asking questions, but I was afraid they would think I was stupid. Instead, they told me that even a senior nurse did not know everything and encouraged me to keep asking questions (Hodges et al.,**2008**).”* If they feel accepted and understood by colleagues, nurses are more motivated to learn, adapt faster to new environments, and are better equipped to handle stress. During the previous COVID-19 pandemic, overwhelming negative news from various media sources made it difficult for people to distinguish reliable information. One nurse said: “*I used to search the literature online and read many articles about the novel coronavirus, especially at the beginning of the epidemic, and I felt that much of what was written was exaggerated (Marey-Sarwan et al.,**2022**).”* Gaining relevant scientific knowledge about the virus restores the belief that the virus can be successfully fought. Similarly, a Chinese nurse improved her professional knowledge and emergency relief ability by closely following the latest literature reports during the pandemic (Li et al., 2021).

### Sense of occupational benefit

According to one systematic review, satisfaction with a career among nurses is positively related to resilience and negatively related to job burnout (Yu et al., 2019). In the research of Hodges et al. (2008), many nurses stated that recognition from doctors, colleagues, and patients boosted their career confidence and was a crucial turning point in their work, “*It was a man in his sixties who was very unstable, and I took care of him for a long time by myself because as everyone else was busy, and then he told me, ‘I feel very safe and comfortable in your care. You are calm and know what to do. ‘I went home that day, and I was thinking, I did it, I can do it! (Hodges et al.,**2008**)*.” Many nurses have reported an increased sense of professional achievement as their patients recovered during the fight against COVID-19, “*I stood in the front line of epidemic prevention with honor and pride, especially when the patients were discharged from the hospital, I felt very contented (Yuan et al.,**2022**).*”

### Social support

Multiple sources of social support for nurses have been described in the literature, including family, team, hospital, and organization. Nurses mentioned that their spouses’ care and understanding, as well as their parents’ encouragement, gave them the courage and motivation to persist (Huang et al., 2021). Team support is also an essential source of resilience. “*My colleagues encourage each other and complain to each other*,” said one nurse, “*The atmosphere in the team is very relaxed and pleasant (Jiang et al.,**2022**).*” Another nurse noted, “*The head nurse went to the front with us, and it made me feel like she had my back (Jiang et al.,**2022**).”* In addition, the hospital administrators provided nurses with the necessary support, such as systematic training for all medical workers and addressing the issue of protective materials in more detail before they went to the frontline (Marey-Sarwan et al., 2022). Furthermore, the hospitals tried to improve the work-life balance for nurses, allowing them more time to rest and spend with their families, which boosted their positive emotions positively (Conolly et al., 2022). Finally, during the public health emergency, the nurses received support from caring individuals of all backgrounds, which helped them recover (Jiang et al., 2020; Li et al., 2021; Marey-Sarwan et al., 2022).

### Self-cognition and regulation

A person’s mentality, beliefs, cognitive abilities, and adaptability affect their attitude toward setbacks and their ability to overcome difficulties. Nurses’ perception of adversity and resilience can help them reflect on challenges and find meaning in them (Hodges et al., 2008). One nurse said: “*When doing the health assessment, there are two ways to deal with stress:* i.e.*, problem-oriented and emotion-oriented. I think I adhere to the former because I can deal with problems calmly, and I am optimistic that any problem can be solved (Huang et al.,**2021**)*.” Another nurse referred to cognitive resilience as more of a motivator to push herself forward, “*I think I am very resilient, I’ve been in ICU for a long time, I’ve been through a lot of traumatic events, and I’ve seen really bad things. Some patients touch you, but other than that, I do not tend to lose my temper at work (Conolly et al.,**2022**).*” Some nurses have also said that their religious beliefs increased their resilience. “*I’m Catholic and must say that prayer helped me handle the situation. It made things less crappy (Ang et al.,**2019*).” At the same time, self-management is also a good way to self-regulate. “*Food can make people happy. During the pandemic, our team often ordered food online (Jiang et al.,**2022**)*.”Another nurse said, “*I read some history books and classical anthologies to relax after work (Jiang et al.,**2022**)*.” Psychological capital is also a crucial protective factor for nurses managing stress, as it can help rebuild their resilience through self-efficacy, positivity, and optimism (Shi et al., 2020).

### Synthesized finding 3: personal growth

The synthesized finding includes two specific categories reflecting personal growth: learn to adapt and self-actualization. These specific categories were extracted from eight articles (Hodges et al., 2008; Mealer et al., 2012; Ang et al., 2019; Jiang et al., 2020, 2022; Shi et al., 2020; Conolly et al., 2022; Yuan et al., 2022).

### Learn to adapt

Adapting to adversity is the first step in personal growth, whether it’s adjusting to work in ECC departments or shifting mindsets during public health emergencies. This shows that nurses have made mental progress through resilience. After going through various difficult and unexpected situations, nurses become more confident and competent, knowing who to ask for help, how to cope with difficult situations, and how to be respected by others (Hodges et al., 2008). One nurse finally said, “*This is my job, this is what I do every day, I know how to do it, and I know what I am doing (Hodges et al.,**2008**).”* During the previous COVID-19 pandemic, nurses have gradually adapted to their work after adjustment. One nurse said, “*It is a heavy day’s work, but I seize the time to rest after work every day so that I can come to work the next day refreshed (Jiang et al.,**2020**).*” Another nurse said, “*Now I do not panic anymore, but sincerely serve the patients and get their recognition and gratitude (Jiang et al.,**2020**)*.”

### Self-actualization

Self-actualization, which is at the highest level of Maslow’s hierarchy of needs, refers to fully developing an individual’s physical and mental talents (Hayre-Kwan et al., 2021). Nurses gain deeper insights into life and career after overcoming adversity and building resilience. One nurse said, “*After this epidemic, I realized that I have many shortcomings. In the future, I need to embrace the belief of lifelong learning, master the latest nursing technology, and fulfill my mission (Jiang et al.,**2020**)*.” Another nurse said, “*I used to feel like I had nothing to do on holidays. After this epidemic, I know that I should seize the free time to invest in myself and make myself more fulfilled (Yuan et al.,**2022**)*.”Another nurse mentioned that she thought more about the meaning of life and understood that health is the most precious thing, making plans to keep exercising in the future to improve her immunity (Li et al., 2021). Some nurses understood the importance of a patient-centered nursing profession and strived to implement humanistic nursing concepts in their work, fulfilling both personal values and professional mission (Hodges et al., 2008).

## Discussion

### Enhancements to resilience strategies still require improvement

The synthesized findings suggest three key factors that impact the resilience of ECC nurses, which are individual cognitive processes and self-regulation, social support networks, and team resilience. While an individual’s cognitive and regulatory abilities are shaped by their past experiences, personality traits, and worldview, social support and team resilience can be influenced by managers. As Richardson noted (Richardson, 2002), developing resilience is a dynamic process that can be deconstructed in response to stimuli and reconstructed in response to changes in stressors and the activation of protective factors. Numerous studies have demonstrated the essential role of social support in fostering resilience (Conolly et al., 2022; Jiang et al., 2022; Marey-Sarwan et al., 2022); however, current nursing management measures remain inadequate (Taylor, 2019). Most strategies aimed at enhancing resilience within the UK National Health Service are predominantly grounded in an individual-centric framework, exhibiting a form of psychocentrism that overlooks the profound impact of social, cultural, and environmental factors. Consequently, this approach partially impedes the cultivation of individual resilience while yielding insignificant research outcomes (Joyce et al., 2018). Some scholars suggest that a holistic approach accounting for organizational culture and team dynamics can lead to a more effective long-term resilience strategy (Conolly et al., 2022).

### The support in multiple dimensions enhances resilience development

Because of the unique working environment and nature of emergency and critical care, organizational support can yield unexpected results. Managers should optimize personnel and provide pre-service training to new nurses, especially in high-requirement departments like emergency and critical care, where new nurses often feel anxious and unfamiliar (Hodges et al., 2008). Pre-service training can alleviate the negative emotions of nurses and facilitate their adaptation to the department environment, enabling them to adapt to their work more quickly and efficiently. Additionally, managers should regularly assess nurses’ skills, train them, and provide them with more opportunities to learn and broaden their horizons to absorb new nursing concepts. Numerous studies have mentioned that the satisfaction of nurses’ desire for knowledge can increase their sense of career benefits to some extent (Hancock et al., 2020). Investment in education, mostly for nurses, could also help improve medical standards. In their study, Jiang et al. (2022) mentioned that giving nurses with clinical work experience a certain degree of clinical decision-making power can also improve their sense of professional benefit and help them understand their professional value. Due to the higher rate of burnout among ECC nurses compared to other departments, hospitals should set up counseling platforms or conduct mental health lectures to encourage nurses to openly discuss their psychological issues and express any stress they may be experiencing. Managers should communicate more, listen attentively, and organize activities to help nurses relax, such as watching movies, listening to music, and playing team games. Cognitive behavioral therapy has also been reported as a positive and efficient mean of improving resilience (Joyce et al., 2018). Other methods, such as debriefing sessions, stress management, resilience training, and mindfulness meditation, can also be used for ECC nurses. Combining multiple intervention strategies has been suggested for greater effectiveness (Joyce et al., 2018).

In terms of management strategy, the long-term nursing management concept solely focusing on patients tends to neglect nurses’ crucial role and significance. According to Drucker (Peter, 2012), enterprises have only one real resource: people. As a concept of people-oriented management, flexible management has gained attention and applications in clinical practice. Studies have shown that such an approach can relieve nurses’ pressure and meet their multi-level needs (Hu et al., 2009). As a fresh management concept, Management By Walking Around (MBWA) emphasizes communication and exchange among managers, the nursing staff, and patients, aiming at understanding nurses’ real needs in on-site management, identifying areas for improvement in clinical practice, solving practical problems, improving nurses’ career satisfaction, establishing excellent relationships between nurses and patients, and promoting rational resource allocation. This approach has been widely applied in intensive care medicine (Jiang et al., 2022). MBWA can enhance communication between ECC departments by promoting multidisciplinary collaboration. A Chinese hospital had tested MBWA in its internal surgery department and found that it significantly reduced adverse events and work errors while improving overall efficiency. These results demonstrate the potential for widespread implementation of ambulatory management in personnel management (Li, 2020).

Regarding team support, we learned that a good team atmosphere and department culture are essential to promote the development of nurses’ resilience, which is consistent with the study of Labrague (2021). Whether it was during the previous COVID-19 pandemic or on a day-to-day basis, the support of colleagues helped nurses through challenging times. However, a poor team atmosphere and colleague bullying can significantly increase negative emotions and damage resilience (Conolly et al., 2022). Therefore, managers should focus on creating a positive department atmosphere, encouraging effective communication among nurses, and organizing activities or training to improve colleague relationships and communication skills. Meanwhile, nurses with high resilience should encourage those with low resilience to work together, promoting resilience through their daily tasks.

Social support also refers to support from family, friends, and patients. Building a healthy nurse–patient relationship has also been reported as an essential source of resilience (Yuan et al., 2022). In the previous study, some nurses mentioned that patient recognition and encouragement increased their forward motivation and confidence (Hodges et al., 2008). Therefore, managers can carry out more lectures and training activities related to therapeutic communication. Simultaneously, they can enhance the guidance of public opinion, disseminate the achievements of outstanding nurses, and elevate the status of nurses in society. Similarly, patients’ defeatist attitudes, death, and other adverse clinical outcomes can also hinder the growth of nurses and even cause vicarious trauma and stress disorders (Yuan et al., 2022). The patients encountered by ECC nurses are usually critically ill, rapidly changing and have high mortality rates. In addition, these departments frequently face public health emergencies such as COVID-19 and adverse outcomes such as patient deaths. Therefore, it is crucial for managers to prioritize psychological counseling for nurses and provide hospice care training. It has been mentioned in the literature that support from hospital administrators is an influential predictor of nurse engagement. Managers can offer further training and develop mentoring programs to encourage nurses (Brykman and King, 2021).

### Team resilience interacts with individual resilience

Team resilience, defined as a team’s ability to recover from adversity or setbacks, is a state of mind among team members based on shared beliefs, common emotions, and motivations (Othman and Nasurdin, 2013). There is an interaction between team resilience and individual resilience: when a team is under pressure or threat, individuals can use psychological resources to extend individual resilience to the team level through interpersonal interactions. Similarly, team resilience affects individuals equally, acting as a protective factor for individual resilience. Consistent with the results of Wang et al. (2023), we found that team resilience had a significant role during the public health emergencies such as the COVID-19 pandemic. Managers should pay attention to the cultivation of team resilience. It has been suggested (Othman and Nasurdin, 2013) that team resilience can be enhanced through fostering a positive team atmosphere, setting leadership goals, improving team learning ability, and promoting information sharing among members. A positive team atmosphere is an important prerequisite for achieving team resilience. Mutual encouragement and support among team members is conducive to extending individual resilience to the team level. The level of resilience of the leader plays a crucial role in determining the resilience of the team. As per the ‘trickle-down’ theory, the development objectives and confidence leaders establish during challenging times directly impact the direction of team progress (Othman and Nasurdin, 2013). Therefore, it is imperative not to overlook training programs to enhance leadership resilience. The ability of a team to learn also influences the development of team resilience. In addition to coping and management methods during adversity and adaptation and growth strategies after, team members should also learn to lower expectations and mentally prepare for adversity. It has been noted that nurses often experience frustration due to a significant gap between their career expectations and the clinical reality they face after starting work (Hodges et al., 2008). To tackle this issue, managers should provide new nurses with comprehensive pre-service training and ongoing guidance on adapting to adversity, identifying problems, learning from them, and adopting a “prepare for the worst, do the best” mindset in their daily practice. In addition, the ability of information elaboration among team members is crucial. This involves effective communication and the integration of diverse perspectives and experiences to facilitate shared learning from adversity. Managers should encourage the exchange of ideas among team members and provide constructive feedback on free opinions to foster the maturity of information elaboration.

### Implications for future public health events

The previous COVID-19 pandemic has given managers new ideas on how to increase nurse resilience during similar public health events in the future, such as addressing material shortages, improving emergency planning and providing accurate media information. Managers should assist nurses in developing multi-dimensional adaptation strategies for public health events, preparing systematic emergency plans, reserving sufficient medical supplies, providing timely training on the latest nursing techniques, guiding nurses in the literature search and information screening, regularly assessing their first-aid abilities, strengthening logistics support and paying attention to their mental health.

## Study limitations

There are several limitations to this review. First, most of the subjects included in the literature are clinical nurses, which is not representative of the entire medical workforce in ECC departments. Therefore, the resilience of head nurses and other leaders needs further discussion. Second, the literature included in this study has a short time span and most of the studies focus on the resilience of ECC nurses during public health emergencies such as the COVID-19 pandemic. Future research should extend the timeline and increase regional diversity to explore the dynamic changes in nurse resilience from a more comprehensive perspective. Finally, the included literature is limited to Chinese and English literature and thus has certain limitations in terms of retrieval. However, this review sheds further light on the factors that influence the resilience of ECC nurses, which care managers can use to develop more viable management plans.

## Conclusion

In total, 12 qualitative studies are included in this review. After further understanding and generalizing the results, nine new categories and three synthesized findings were summarized, and the ConQual system scores of all the integrated results were moderate. Our results show that the resilience of ECC nurses is influenced by a variety of factors, with individual cognition and regulation, social support, and team resilience being particularly significant. Therefore, nursing managers should implement reasonable measures to improve the mental health of nurses and promote the development of resilience through improvements in work environment, departmental atmosphere, personnel optimization. In addition, nurses should be encouraged to fully utilize their self-worth and uphold their professional mission while ensuring safety. In the future, carefully planned and robust intervention studies should be conducted to validate the efficacy of resilience-enhancing strategies while taking into account the evolving trajectory of long-term resilience development among nurses.

## Data availability statement

The original contributions presented in the study are included in the article/supplementary material, further inquiries can be directed to the corresponding authors.

## Author contributions

SL: conceptualization, methodology, formal analysis, writing-original draft, and writing-review and editing. YZ: conceptualization, methodology, writing-original draft, and writing-review and editing. PH and YL: conceptualization, methodology, formal analysis, and writing-review and editing. JJ and YZ: methodology, formal analysis, and writing–review and editing. All authors contributed to the article and approved the submitted version.
